# Supplementary material for: Quantifying the varying harvest of fermentation products from the human gut microbiota
Source: Cell. Author manuscript; Available in PMC 2025 Oct 27. (PMC12556654; doi:10.1016/j.cell.2025.07.005)
Supplement: MMC1 [file NIHMS2099869-supplement-MMC1.pdf]

Cell, Volume *188*

Supplemental information

Quantifying the varying harvest of fermentation products from the human gut microbiota

Markus Arnoldini, Richa Sharma, Claudia Moresi, Griffin Chure, Julien Chabbey, Emma Slack, and Jonas Cremer

**Observable**	**Symbol**	**Species average**	**Cross-species range**	**molecular weight**	**carbon per molecule**	**combustion enthalpy (*H_i*)***
Carbohydrate uptake	u_{carbs}	14.1 mmol glucose equivalents/g	6.1-24.1 mmol glucose equivalents/g	180.2 g/mol	6	
Total excretion	e_{tot}	26.7 mmol/g	$8.0 - 19.0$ 8.0-19.0 mmol/g			
Acetic acid excretion	e_{act}	11.8 mmol/g	0.1-27.2 mmol/g	0.88 g/mol	2	0.88 kJ/mol
Butyric acid excretion	e_{but}	1.2 mmol/g	0.0-8.7 mmol/g	2.18 g/mol	4	2.18 kJ/mol
Formic acid excretion	e_{for}	7.3 mmol/g	1.8-20.6 mmol/g	0.26 g/mol	1	0.26 kJ/mol**
Lactic acid excretion	e_{lac}	4.2 mmol/g	0.0-11.4 mmol/g	1.38 g/mol	3	1.38 kJ/mol
Propionic acid excretion	e_{pro}	2.3 mmol/g	0.1-13.6 mmol/g	1.55 g/mol	3	1.55 kJ/mol
Succinic acid excretion	e_{suc}	1.9 mmol/g	0.0-7.1 mmol/g	1.51 g/mol	4	1.51 kJ/mol
