# Supplementary material for: Quantifying the varying harvest of fermentation products from the human gut microbiota
Source: Cell. Author manuscript; Available in PMC 2025 Oct 27. (PMC12556654; doi:10.1016/j.cell.2025.07.005)
Supplement: MMC3 [file NIHMS2099869-supplement-MMC3.zip › cell_00014072_Supplemental Data2.html]

Bokeh Plot


**Please note:** This interactive figure is also available via the repository of our study, accessible at
https://cremerlab.github.io/fermentation\_products/
