# Supplementary figures and images for: Quantifying the varying harvest of fermentation products from the human gut microbiota
Source: Cell. Author manuscript; Available in PMC 2025 Oct 27. (PMC12556654; doi:10.1016/j.cell.2025.07.005)

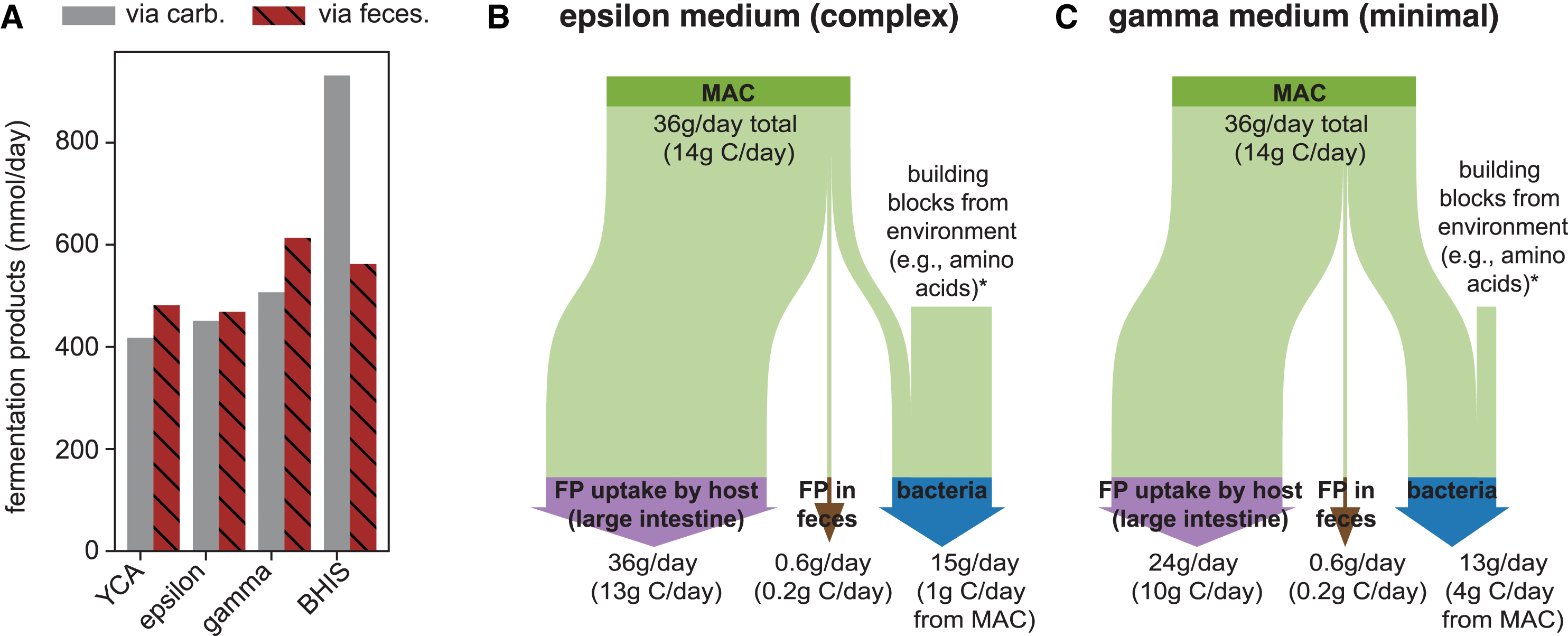

Supplement: figs5 — Figure S5. Variation in estimates of fermentation product harvest with media, related to Figure 2 (A) Estimation via feces and carbohydrates for the British reference scenario, using average per-biomass uptake and excretion rates measured for the different media (Figure S3). The resulting daily release of fermentation products is comparable across media, except for the estimation via carbohydrates in BHIS. This is likely an artifact of the unknown carbon sources in this medium, the consumption of which we are unable to measure, leading to an underestimation of the per-biomass carbohydrate uptake rate. (B and C) Flow diagrams showing the resulting carbohydrate and carbon flow along the large intestine when using characteristics measured in gamma and epsilon media, respectively. Diagrams similar to the one shown in Figure 2E for YCA medium, but, as fewer strains grew in these media, we used simple rate averages and did not weight rates by biomass abundance. An internally consistent flow diagram for BHIS was not constructed due to the underestimation of carbohydrate intake in this medium. [file NIHMS2099869-supplement-figs5.jpg]

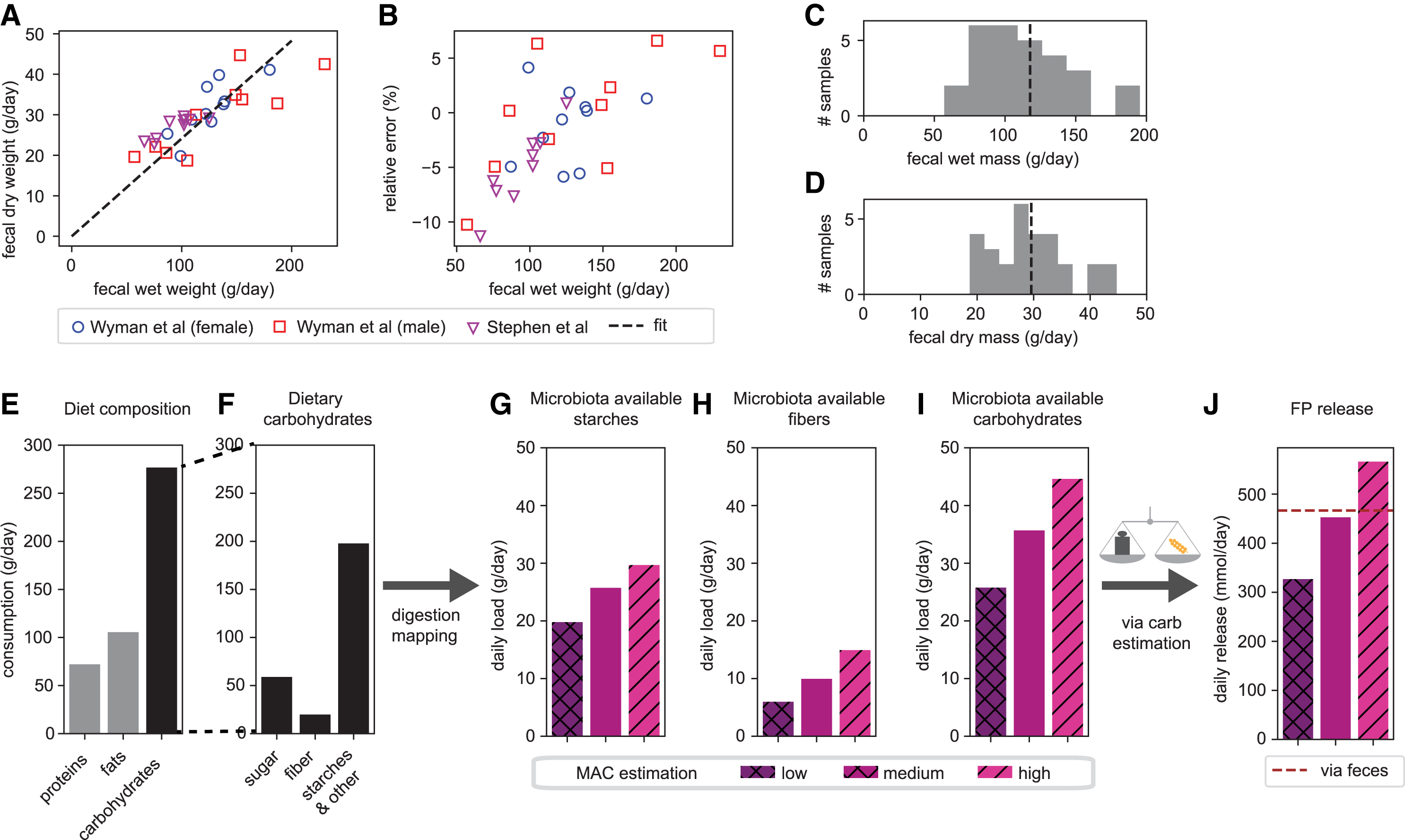

Supplement: figs4 — Figure S4. Characteristics of the British reference diet and variation of the microbiota-available carbohydrates with different efficiencies of complex carbohydrate digestion, related to Figure 2 To describe the relation between fecal dry and wet weight in people consuming a British reference diet, we use data from Wyman et al.25 where both values are reported. (A) Linear regression (dashed line) assuming a fixed fraction of dry weight per wet weight describes the relation well. (B) Relative error of the linear regression model. Errors smaller than 10% confirm the linear model. (C and D) Histograms of fecal wet and dry weight to illustrate their substantial variation. (E and F) Diet composition of the British reference diet as provided in the anual report of the National Food Survey Committee from 1976.27 (G–I) Digestibility of starches changes with the type of food consumed and the cooking method. The ability to digest different types of fibers also depends on the specific metabolic capabilities of the microbes present. To illustrate the effect of this variation, we use three different mappings from dietary carbohydrates to microbiota-available carbohydrates (low, medium, and high), accounting for the large variation in fiber digestibility (see Data S1, section 4). These assumptions lead to varying amounts of microbiota-available starches and fibers, the sum of which is the total amount of microbiota-available carbohydrates. The medium case shows the best-established mapping for the British reference diet and is used in the main text. (J) The different mapping assumptions lead to different estimates of fermentation product release. Parameters used for MAC mapping follow typically observed ranges of starch content and fiber digestion, with 15% starch passage and 75% microbial fiber digestion for the high case (poor complex carbohydrate digestion along the upper digestive tract), 13% starch passage and 50% fiber digestion for the medium case, and 10% starch passage a [file NIHMS2099869-supplement-figs4.jpg]

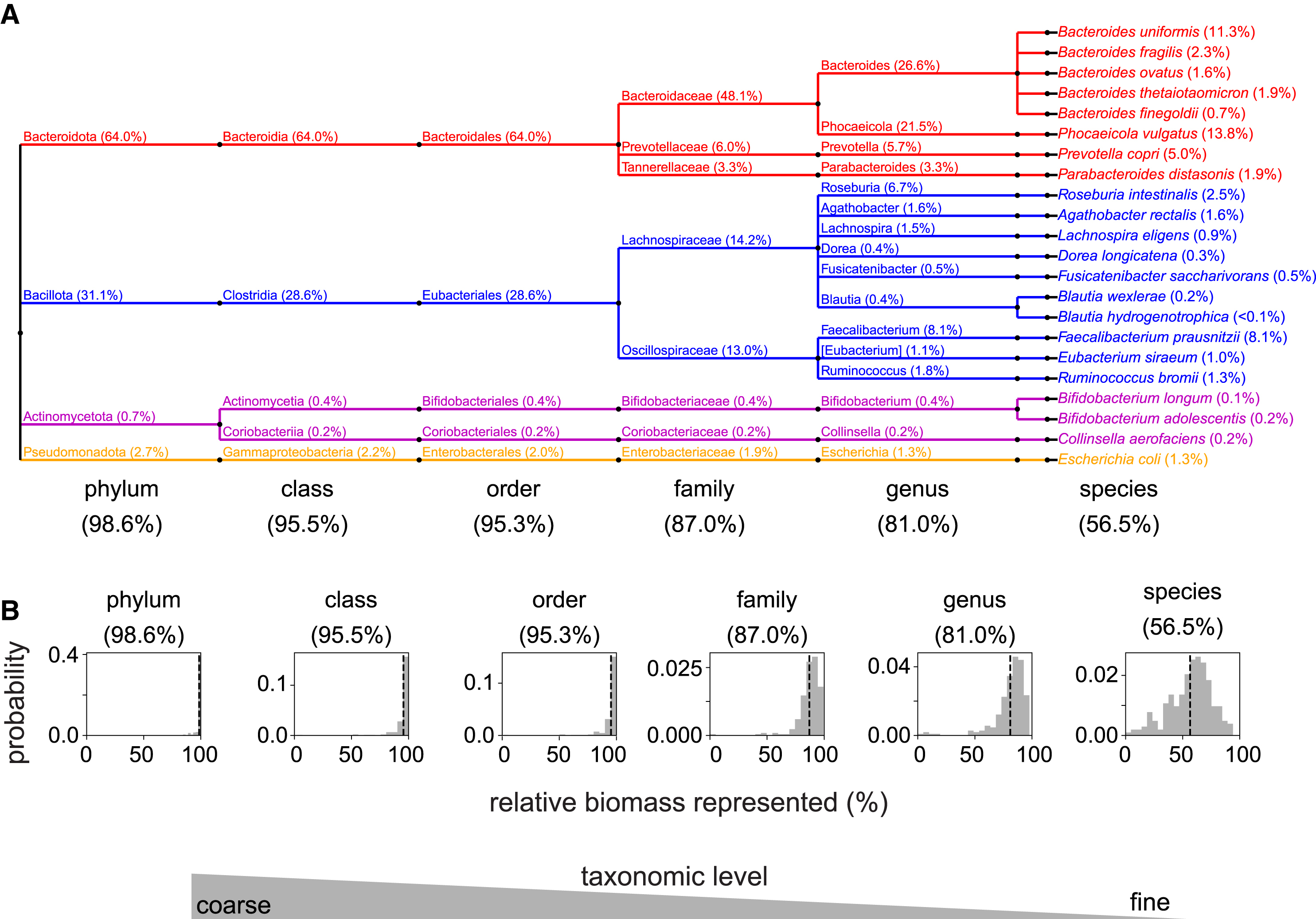

Supplement: figs2 — Figure S2. Taxonomic relation and biomass coverage of experimentally characterized strains, related to Figure 1 (A) Phylogenetic tree of 22 highly abundant human gut microbiota strains we characterized. Branch labels indicate the names of different taxonomic groups. Numbers show the average coverage these strains represent on the species and higher taxonomic levels in a collection of 219 microbiome samples from healthy individuals.21,22 For example, the 22 strains account, on average, for 59.6% of all bacterial biomass on a species level, 83.7% on a genus level, etc. (B) Breakdown of the sample-to-sample variation of this coverage. Coverage for additional studies is shown in Data S2. [file NIHMS2099869-supplement-figs2.jpg]

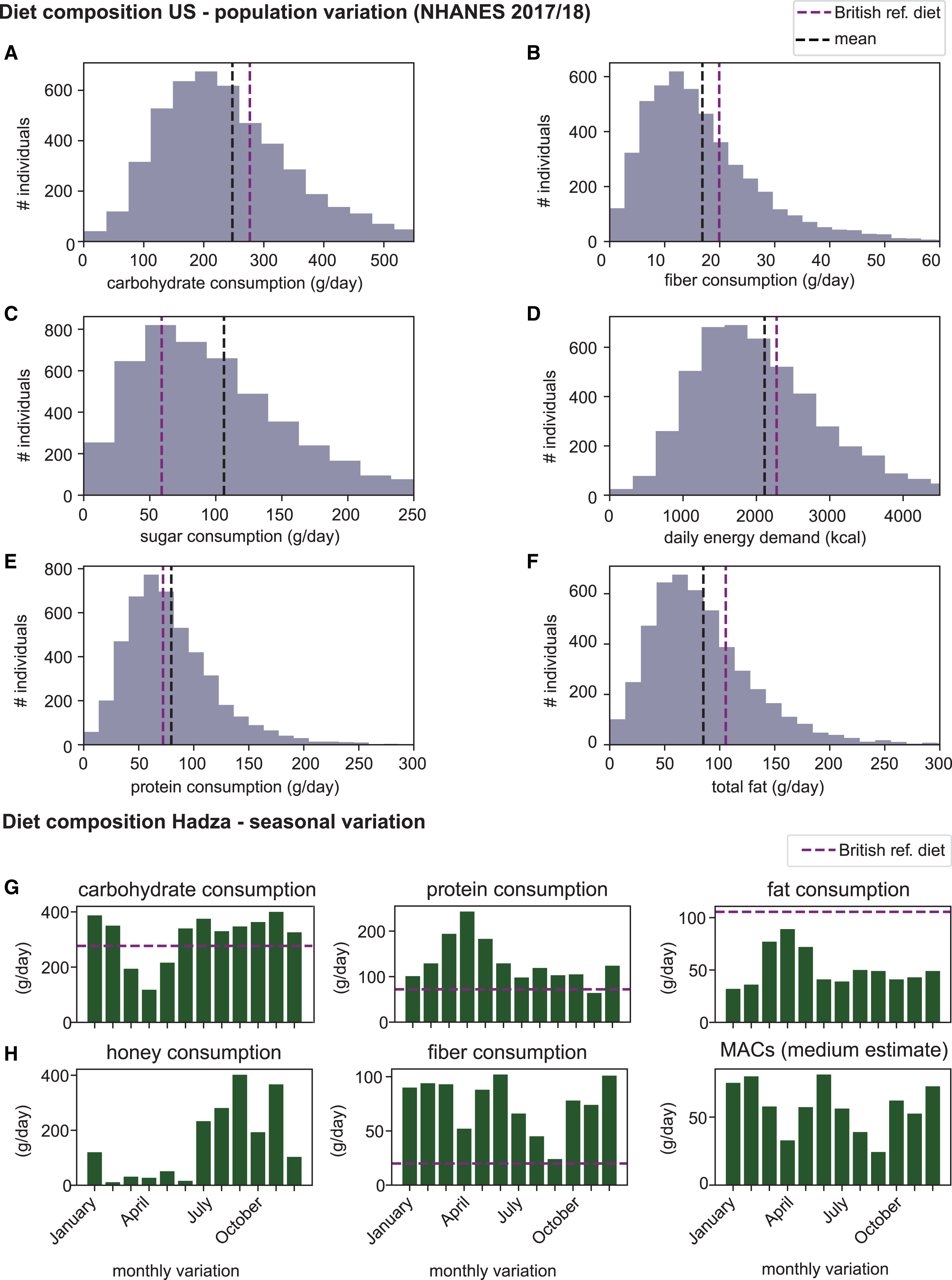

Supplement: figs7 — Figure S7. Major dietary components for the US and the Hadza populations, related to Figure 4 These data underlie the estimates for microbiota-available carbohydrates and total fermentation product release shown in Figure 3. (A–F) Distribution of different dietary characteristics across the US population based on the NHANES 2017/2018 cohort.33 Black dashed lines indicate means of distributions. (G and H) Per-capita breakdown of major diet components based on food collected by a group of Hadza people, as reported by Pontzer and Wood.37 Purple dashed lines in different panels indicate corresponding numbers for the British reference diet (Figure S4). [file NIHMS2099869-supplement-figs7.jpg]

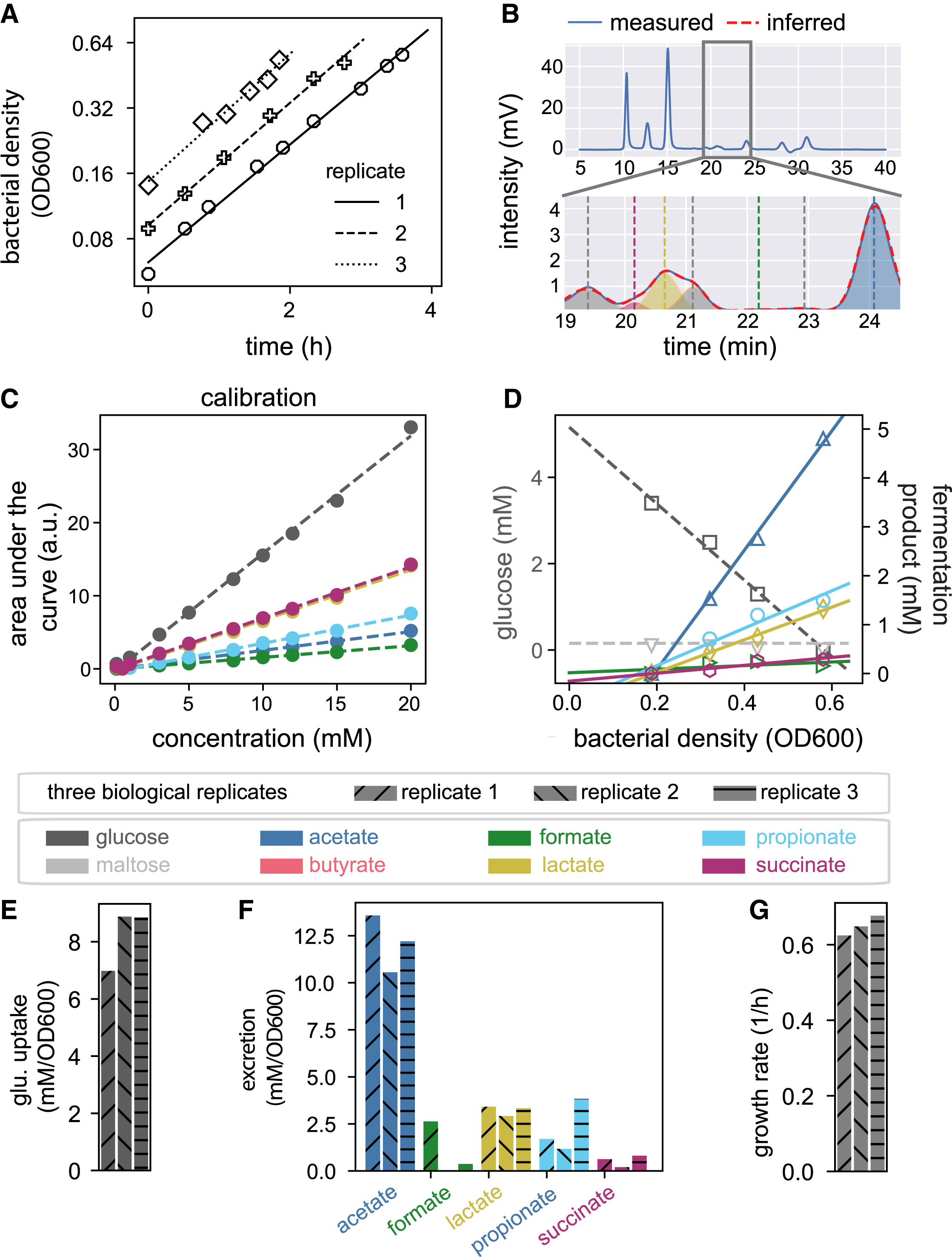

Supplement: figs1 — Figure S1. Experimental workflow to determine the per-biomass excretion and consumption rates of metabolites, related to Figure 1 (A) Optical densities (OD600) were measured over time and samples were taken at different OD600 values during exponential growth (symbols). Culturing in two steps included a pre-culture phase before sample taking to ensure steady growth. Different lines show three independent biological replicates. (B) These samples were then analyzed for metabolites using HPLC. Peaks for specific metabolites were identified, computationally isolated, and the areas under the curves they cover were quantified using a Python-based data analysis pipeline we have developed.14,73 (C) Obtained peak areas were subsequently compared with the shown component-specific standard curves to determine their concentrations. (D) Linear fits describing the change of these concentrations with optical density, as expected for steady growth, were then used to calculate the per biomass production and consumption of different metabolites. (E and F) Per-biomass excretion of fermentation products, {ei}, and uptake of glucose, u, for three biological replicates. (G) Exponential growth rates μ of three biological replicates, obtained by a linear regression on the log-transformed OD data shown in (A). From these numbers, excretion and uptake per time can also be calculated. Exemplary data shown here for B. theta growing in YCA medium. Similar plots for other species and growth conditions are available via the GitHub repository (folder hplc_measurements). [file NIHMS2099869-supplement-figs1.jpg]

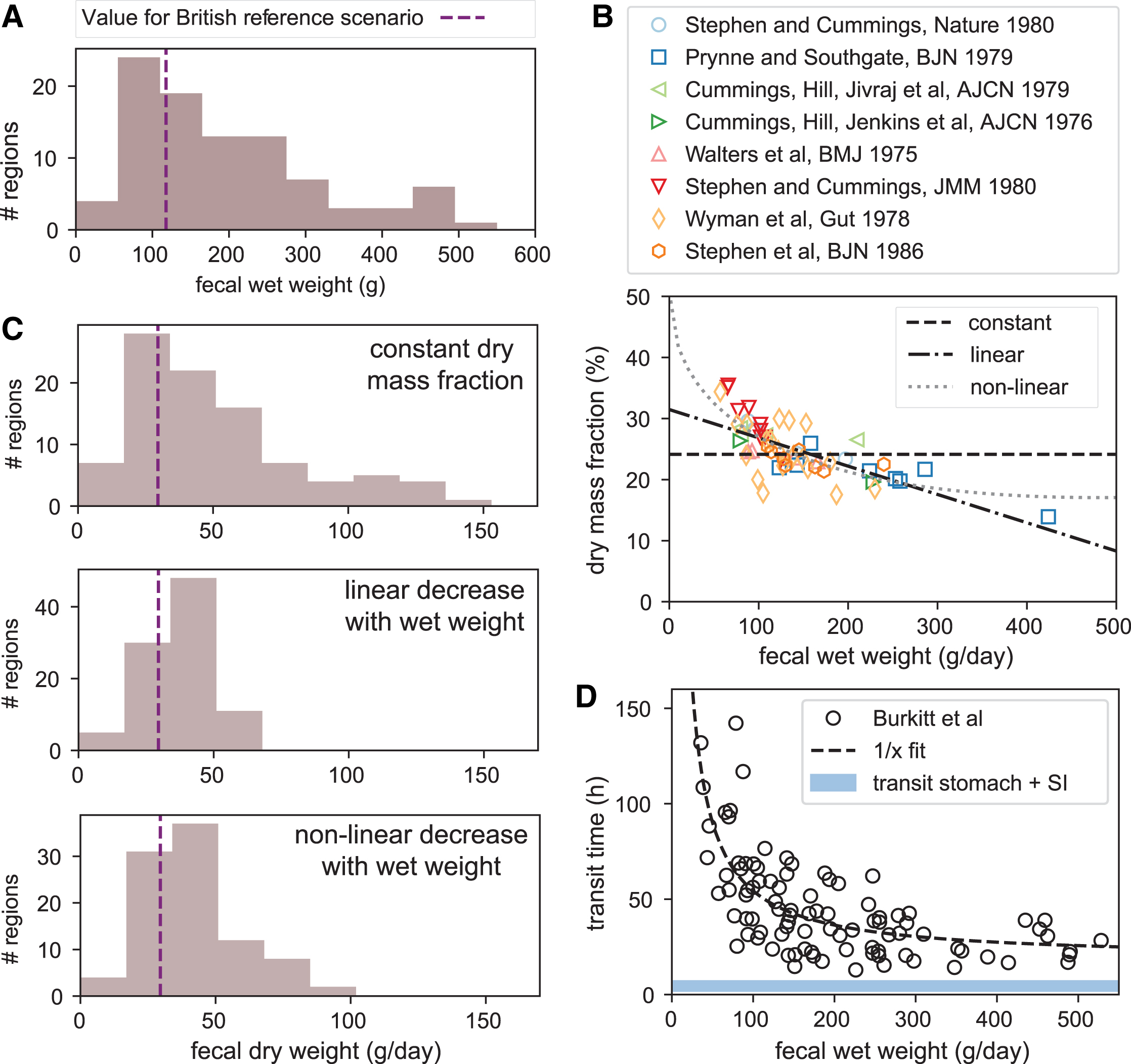

Supplement: figs9 — Figure S9. Global variation in fecal weight and variation of fermentation product release with fecal water content, related to Figure 4 (A) Variation in fecal wet weight in humans of various backgrounds and lifestyles, as reported in Burkitt et al.38 (B) Shown data (markers) were collected from different studies that measured fecal wet and dry weight for different cohorts and dietary compositions.17,25,26,40–44 When the variation in fecal output gets larger, water content in feces increases. Therefore, the assumption of a constant ratio of dry weight per wet weight we have been using for the British reference case (Figure S4) leads to substantial errors in describing the observations (“constant,” dashed line). To account for larger variations in water content, we formulated two additional models to fit the data. In the linear model, the dry mass fraction (αdw) decreases linearly with fecal wet weight (“linear,” dotted dashed line, αdw=1-4.63*Mfeces,wet+0.69. In the non-linear model, the dry mass fraction (αdw)decreases approximately linearly with fecal wet weight when wet weight is low, but it hardly changes anymore when fecal wet weight is high (“non-linear,” dotted line, αdw=1-(0.49+0.03⋅1/√g*Mfeces,wet-0.0007*Mfeces,wet⋅1/g). Both models describe the trends of the available data well. We expect the dry mass content to decrease further with higher fecal wet weight as very high weights are likely caused by a higher water content bound to fibers in feces. However, as no parallel measurements of fecal dry and wet weights are available for very high weights, the non-linear model provides a well-supported upper bound of fecal dry weight. (C) With these different models, we then estimated the fecal dry weight for the data reported in Burkitt et al.38 Notably, the variation in fecal dry weight decreases substantially when accounting for the adjustment in dry mass fraction. For the estimations discussed in the main text, we used the non-linear model. As such, these number [file NIHMS2099869-supplement-figs9.jpg]

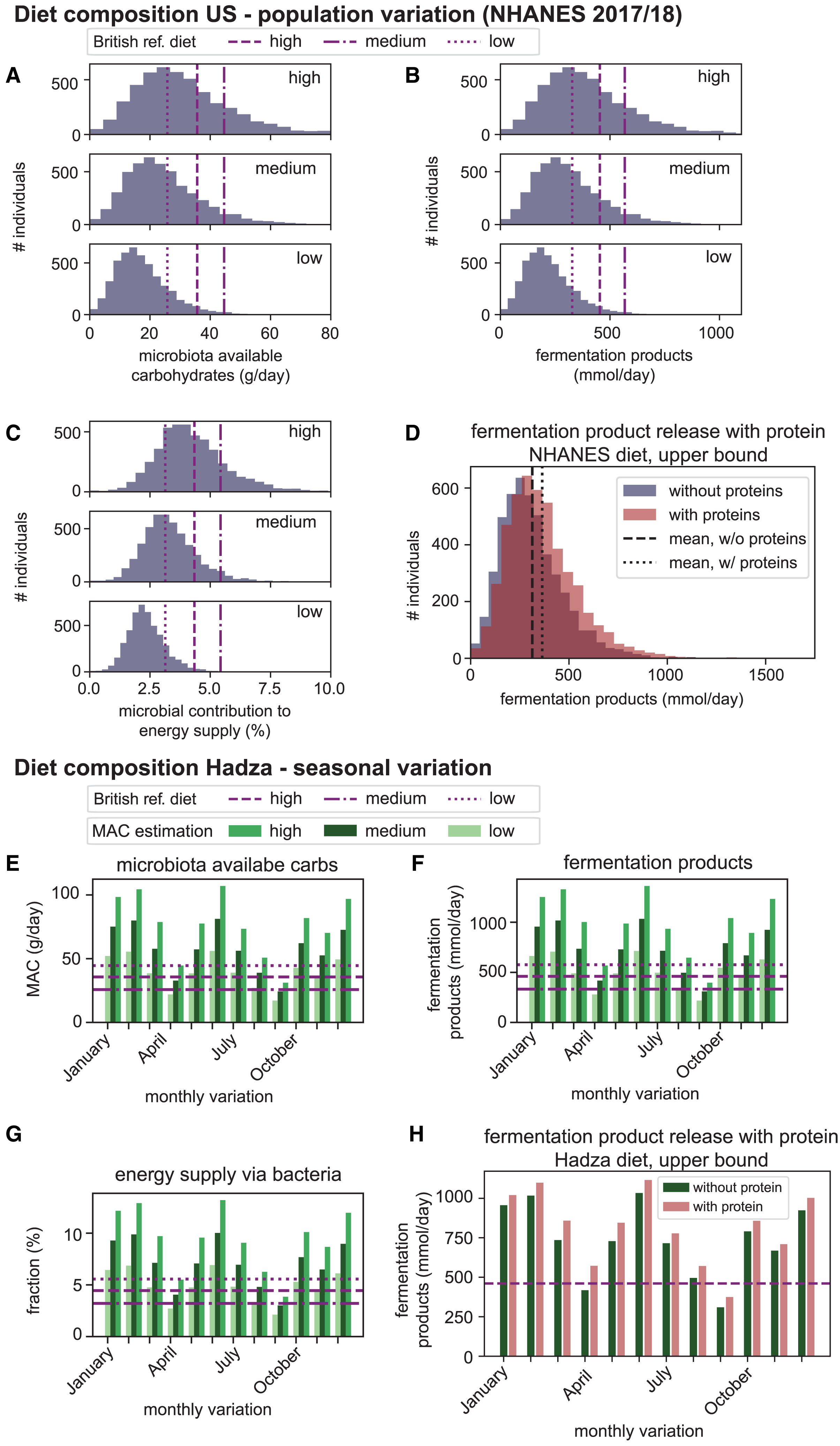

Supplement: figs8 — Figure S8. Variation of fermentation product release with diet, given different assumptions of carbohydrate digestion, related to Figure 4 We analyzed the change in microbiota-available carbohydrates for different diets to estimate the total fermentation product release when changing the assumptions on carbohydrate degradation along the upper digestive tract within observed ranges (see Figure S4; Data S1, section 4 for further discussion of the mapping). (A–C) Changes in microbiota-available carbohydrate, total fermentation product release, and microbial contribution to the daily energy demand with reported variations in carbohydrate consumption reported for the US NHANES 2017/2018 cohort. (D–F) Changes in microbiota-available carbohydrates, total fermentation product release, and microbial contribution to the daily energy demand with the reported month-to-month variation of per-person carbohydrate amount in food in a group of Hadza people. (G) Fermentation product release for the NHANES cohort with (red) and without (blue), accounting for the fermentation of dietary proteins. (H) Fermentation product release for the Hadza people with (red) and without (green), accounting for the fermentation of dietary proteins. Changes in (G) and (H) are small, even for the used upper bound estimation of bacterial protein digestion (Data S1, section 5). Following data for the British reference scenario (Data S1, section 4), parameters used to describe the mapping between consumed and microbiota-available carbohydrates are 15% starch passage and 75% fiber digestion for the high case, 13% starch passage and 50% fiber digestion for the medium case, and 10% starch passage and 30% fiber digestion for the low case. In the main text, data for the medium case are shown. Purple lines indicate corresponding estimations for the British reference diet. [file NIHMS2099869-supplement-figs8.jpg]

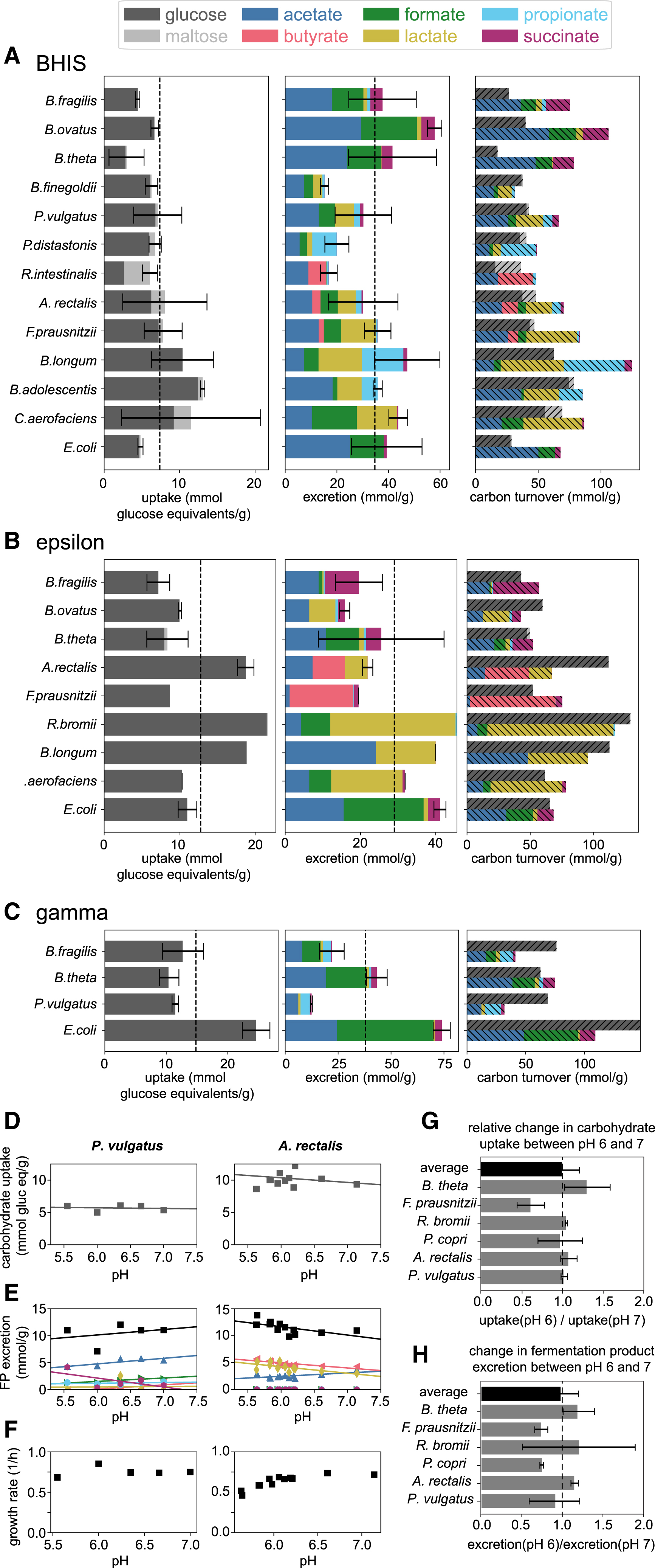

Supplement: figs3 — Figure S3. Uptake and excretion characteristics for growth in different conditions, related to Figure 1 (A–C) Sugar uptake and fermentation product excretion per biomass for growth of different gut species in three different media (BHIS, γ, and ϵ). Complexity of medium composition decreases from BHIS (A) to YCA (Figures 1D–1F) to epsilon (B) and gamma medium (C). See STAR Methods for details on media composition. With decreasing medium complexity, fewer of the 22 strains can grow. Strain-by-strain visualizations of growth curves, fermentation product excretion, and sugar uptake for all media conditions and biological replicates can be found in the paper’s GitHub repository. Error bars in (A)–(C) denote SD of three biological replicates. (D–H) Sugar uptake and fermentation product excretion per biomass for growth at different pH values of 6 abundant bacterial species representing the most abundant genera and families. pH variation of carbohydrate uptake per dry mass (D), fermentation product excretion per dry mass (E), and growth rates (F) for P. vulgatus and A. rectalis, respectively. Black squares and lines in (E) indicate total fermentation product excretion. Linear trends with pH were fitted to determine the relative change in uptake (G) and excretion (H) between pH 6 and 7. Error bars denote error in slope based on linear fit. Data for B. thetaiotaomicron and A. rectalis from Cremer et al.14 [file NIHMS2099869-supplement-figs3.jpg]

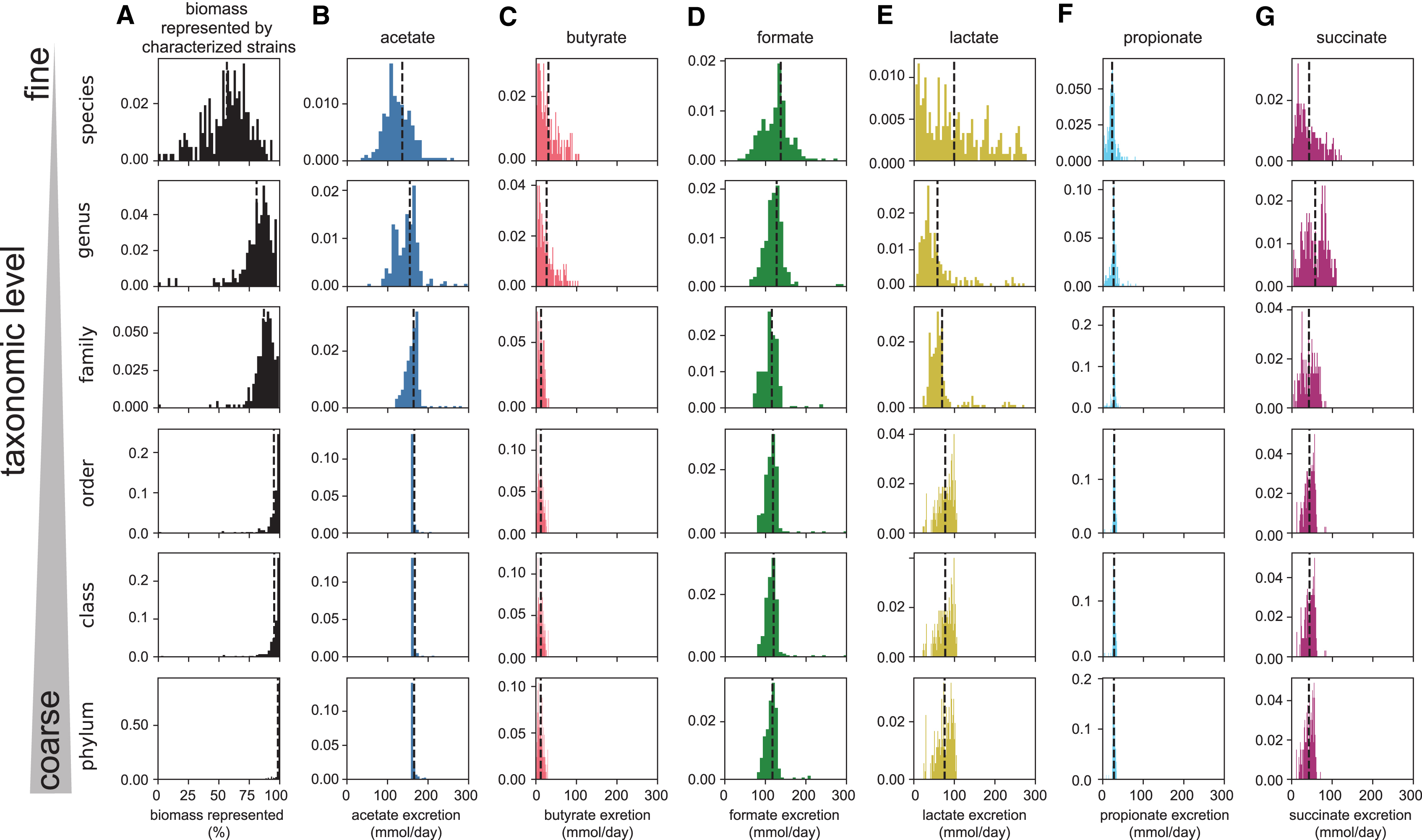

Supplement: figs6 — Figure S6. Representation and analysis of fermentation product release on different taxonomic levels, related to Figure 3 To decide which taxonomic level to use for our analysis, we probed how much of the abundance in the healthy human gut microbiome21,22 is represented by the 22 strains we have characterized experimentally. Covered abundance changed with the taxonomic level we chose for our analysis, with a coarser level leading to higher coverage (gray histograms). For example, on the species level, the characterized strains represent an average of about 60% of microbial biomass, whereas, on the phylum level, the strains represent, on average, more than 90% of bacterial biomass. Thus, choosing a coarser level will lead to a higher representation by experimentally characterized strains, and we need to make fewer assumptions on how to describe the uptake and excretion rates of experimentally non-represented bacterial biomass. On the other hand, we also lose resolution in describing the production of specific fermentation products when choosing a taxonomic level that is too coarse. Particularly, the metabolic behaviors of strains belonging to the same taxonomic group might differ substantially at coarser taxonomic levels. For example, consider the production of succinate, which is produced in substantial quantities by some, but not all, species of the phylum Bacteroidota. When using average uptake and excretion rates, including all experimentally characterized strains belonging to the Bacteroidota phylum, we will lose possible differences in succinate release. As the best compromise, we thus chose the genus level for our analysis in the main text, as biomass coverage is already very high, while we avoid too much averaging out of strain-level differences. See Data S1, section 2 for further discussion. [file NIHMS2099869-supplement-figs6.jpg]
